# An Explanation for Repetitive Motor Behaviors in Autism: Facilitating Inventions via Trial-and-Error Discovery

**DOI:** 10.3389/fpsyt.2021.657774

**Published:** 2021-07-01

**Authors:** Catherine L. Caldwell-Harris

**Affiliations:** Department of Psychological and Brain Sciences, Boston University, Boston, MA, United States

**Keywords:** autism, evolution, repetition, invention ability, special interests

## Introduction

Restricted/repetitive behaviors is a core diagnostic criterion for autism. Motor repetitions, referred to as “lower-order,” include self-stimulation, hand flapping, twirling, repeating phrases, manipulating objects, banging toys together, and repeatedly pushing buttons (1). Also included in the broad category of restricted and repetitive behaviors are cognitively advanced, “higher-level” behaviors, rituals and circumscribed or restricted interests (2, 3). These types of repetitive behaviors superficially look different. For instance, repetitive motor behaviors can cause self-injury (e.g., head banging) and interfere with learning and family life (4). In contrast, some scholars and autistic individuals themselves have relabeled restricted interests as “special interests.” This follows the strength-based approach of the neurodiversity movement, including advocacy by autism individuals themselves [e.g., (5)]. Special interests can readily be understood as on a continuum with neurotypical hobbies, workplace specialization and the research interests of scientists (6, 7). Theorists have long noted that intense interests of persons with autism can be precursors to scientific discovery and achievement [e.g., (8–10)]. No comparable adaptive function has been proposed for repetitive motor actions. This is the purpose of the current paper.

What causes repetitive motor behaviors? At the level of genetic alterations, developmental heterochrony is one plausible mechanism (11). Many features of autism could result from extending the longevity of motor repetitions beyond early childhood into the juvenile years and adulthood (11, 12). Motor reflexes are integral to survival in infancy, but gradually come under voluntary control with maturation of the cortex in the first 2 years of life (13). Motor repetitions resembling those observed in autistic individuals are common in children during early childhood, but disappear by age 4–6. This is plausibly one factor for why autism is hard to diagnose before ages 3–4.

Other mechanisms for dysfunctional motor repetitions have been proposed. Parts of the brain involved in regulating motor systems have demonstrated abnormal functioning in autism. For example, the cerebellum has long been pinpointed as a likely candidate for both motor and cognitive deficits [e.g., (14)]. Striatal dysfunction has also been implicated, which is especially intriguing give that striatal circuits are important for both cognitive and social abilities (15). Whether these or other brain systems could have been altered as part of the heterochrony proposed by Crespi (11) is unknown.

Do motor repetitions serve any adaptive function, or are they only maladaptive? Motor routines allow persons with autism, especially young children, to avoid on-going social demands (16). The most frequently cited function is that motor repetitions are calming in the face of social and other stressors [e.g., (17)]. These stressors include difficulty in predicting ongoing events, resulting from weak central coherence, executive function challenges and social deficits. Self-regulation as a cause of motor repetitions is mentioned by autistic individuals themselves [e.g., (18)]. It is also plausible because humans commonly regulate both hyper- and hypo-arousal with scratching, hair/beard twirling and finger tapping; animals groom or scratch as calming strategies. Motor repetition is also heightened for animals and humans in deprived environments.

A drawback of the stress-reduction explanation is that motor repetitions in autistic individuals are not invariably or even usually associated with distress (12). Minimally verbal children often engage in repetitions with focused determination, ignoring adults' bids for attention. In addition, repeated motor behaviors observed in some autistic children can be goal directed and pursued with interest and focus. In this way, they resemble the engagement observed for pursuit of special interests, as in the mechanical tinkering of the young Isaac Newton or young Thomas Edison (8, 9).

My focus in the current paper is on motor repetitions and repetitive object use, but the ideas I present are also relevant to related motor actives, such as self-stimulatory behavior, verbal repetitions and visual repetitions [inspecting toys in an unusual manner, see taxonomy in Harrop et al. (1)]. However, the umbrella category of restricted/repetitive behaviors include sensory aversion, sensory seeking, and behavioral inflexibility (insistence on sameness). I believe these behaviors require a different explanation [see (19) for a unified approach]. For example, auditory and olfactory acuity were adaptive in the ancestral environment when humans seeking shelter needed to determine if a predator was in a cave (10).

Theorists have commented that the field needs more theories regarding the function of repetitive motor behaviors [e.g., (1, 12, 20, 21)]. I outline here an adaptive function that is plausible from the standpoint of human evolution:

*Retaining motor repetitions into childhood and adulthood allowed repetitive motor sequences to fuel trial-and-error discovery*.

In Temple Grandin's memorable words, “Who do you think made the first stone spear, it wasn't the social yakety-yaks sitting around the campfire” [(22); see also (23), p. 122]. Motor routines may indeed be calming or self-regulating, but they may also be pursued for their own interest and rewards. They may be pursued for the delight in the interesting variation which can result from minor deviations. What would happen if I cut this rock at an angle—would it make a sharp point? When results are interesting, and especially if predictions are correct, trial-and-error tinkering can be reinforced by the dopamine reward system (24).

During much of human prehistory, technological advances in tools, weapons, fishing craft, and shelter construction may have been fueled in part by repetitive motor explorations (and systematic observation) of sticks, stones, plants, and in rivers/lakes (10, 25). Note that autistic individuals do not need themselves to always recognize the usefulness of a novel configuration. The “aha” moment may occur in the brains of observers. Usefulness of the invention can help group members to be indulgent toward the socially-nonconforming autistic person.

I will refer to this hypothesis as *motor tinkering for trial-and-error discovery*.

The trial-and-error work of scientists fits with our intuitions about inventions (8), but the adaptive function of simple motor repetitions is less obvious. Cziko (26) documented how trial-and-error exploration of objects is a key mechanism in invention in diverse species. Genetically-specified motor programs, combined with variation to fit a specific environment, produce spider webs, beaver dams and bower displays. Crows' fixed action patterns can result in tool use (27). Human tinkering with objects also plausibly led to cultural discoveries for building shelters, creating weapons and detoxifying food (28). From this perspective, there would have been substantial selection pay-offs for a phenotype in which the repetitive behaviors of early childhood were maintained into the juvenile period and adulthood.

## Evaluating “Motor Tinkering for Trial-and-Error Discovery”

### Consistent With Other Evolutionary Hypotheses

The hypothesis is consistent with Crespi's (29) characterization of autism as a disorder of imbalanced intelligence, that is, a mix of enhanced and impaired abilities. During natural section, increased analytical intelligence likely reaped fitness benefits, allowing extensive exploration in the fitness landscape. One plausible result is diverse phenotypes with pockets of enhanced ability co-existing with deficits. Motor tinkering that could result in novel useful configurations is plausibly a phenotypic variation resulting from these fitness pressures.

### Bridge Between the Motor Repetitions and Restricted Interests

Although motor repetitions and narrow interests are grouped under the umbrella terms restricted/repetitive behaviors, their similarities are not obvious. I propose that repetitive motor behaviors are part of the engine that fuels systemizing of the natural world, leading to pattern extraction, if-then rules, and technological discoveries [as discussed by Baron-Cohen (8)].

An example of a bridge to circumscribed interests can be observed in cases where a motoric ritual is also a child's special interest. In videos of his young autistic son, Love (30) documents that habitual stair-climber Frumpkin must spend 15 min traversing any newly encountered staircase. Stair-climbing is both a motor preoccupation and an intense/restricted interest. But Frumpkin had other motor interests that shared a family resemblance structure with stair-climbing. In a park, he discovered picnic tables arranged to allow a complete circle to be made on table-tops and their benches. He then obsessively circumnavigated the table-tops, resistant to parental intervention. Frumpkin's father noted that Frumpkin “mixes it up”—he doesn't walk in the same manner each time, as if he is observing the variation that results from slight deviations in his path.

Frumpkin's attraction to both staircases and table-tops suggests a more abstract underlying interest: He is trying to systematize walkable, raised, man-made surfaces. From the perspective of human technological development, tables and staircases are extremely interesting. Staircases are complex inventions whose construction is non-obvious. Generations of cultural evolution were required for invention, refinement and modern-day craftsmanship. One could conceive of Frumpkin as a future engineer obtaining sensory-motor schemes that could facilitate future innovations in design of raised wooden-surface walkways.

### But: Many Motor Repetitions Are Not Geared Toward Discovery

Many behaviors in the broad category of restricted/repetitive behaviors are maladaptive and would have harsh fitness consequences during human evolution, as well as today. The plausible cause of this is that heterochrony is a blunt instrument, given that natural selection is not goal-directed. Susceptibility alleles for retaining repetitive motor repetitions beyond early childhood would need to be maintained in the population via balancing selection (31), with many individuals with autism making no fitness-enhancing discoveries.

Open questions about this include:

- What proportion of individuals with the autism phenotype must deliver fitness-enhancing discoveries in order for genes for autism to spread or be maintained in a human population?
- In what cultures or environments do repetitive motor behaviors lead to discoveries? Contemporary inventors with autism who also have high analytical intelligence (such as Elon Musk) can gain the social status consistent with reproductive benefits. But my hypothesis rests on natural selection in the ancestral environment, where discoveries included stone tools and other physical artifacts. Are there any contemporary societies in which motor tinkering leads to useful discoveries?

### Heuristic Value of the Motor Tinkering Hypothesis

I propose that motor routines are information-seeking and in part driven by the reward of discovery, similar to intense interests and scientific discoveries. However, the “blunt instrument” of heterochrony and a neural system with revved-up motor programming means that many minimally verbal autistic children perform unvaried motor routines for hours a day, disrupting family life (21).

One therapeutic approach is that adults can model for children how to vary their motor routine, but in a direction that is inherently rewarding for the child. Parents and therapists can observe and participate in their child's motor activities, drawing on techniques in JASPER (32) and Floor Time Play Therapy (33). While observing, the adults use their own systemizing and prediction skills to plan a variation in the motor routine that could result in a pleasing or interesting result. The adults then assist the child in moving toward a rewarding variation, or directly model such a move. For example, for a motor routine like table-circling, the adult could tap on the table at a predictable spot, or purposefully flip over a strategically placed object (or a more spectacular result could be planned). The pay-off for the child is the inherent interest of a new event. But the behavior being reinforced is modifying the repetitive motor actions in the direction of variation and flexibility.

### Alternative and/or Complementary Approaches

Two comprehensive proposals about systemizing and patterning in autism were published after the first draft of this paper. Baron-Cohen's book *The pattern seekers: How autism drives human invention*, connects his decades of work on systemizing to an evolutionary pay-off, invention. Baron-Cohen notes that autistic individuals excel at if-then reasoning, and argues that if-then reasoning allowed humans to become the top inventers in the animal kingdom. My hypothesis is complementary but distinct: Motor tinkering fosters trial-and-error discovery. Motor repetitions with slight variations (tinkering) can involve if-then conceptualization, as follows: “IF I pile my rocks his way, THEN they will form a new configuration, a vertical surface.” Note that the motor behaviors of many animals amount to tinkering, as when a beaver uses trial-and-error to configure a dam. The motor-tinkering theory is otherwise similar to Baron-Cohen's approach, in that both emphasize invention as the adaptive benefit of repetitive and restricted behaviors.

Crespi (19) proposes that what is common across diverse autistic symptoms is the concept of pattern. For example, pattern seeking leads to high systemizing and interest in STEM disciplines. Highly tuned pattern perception is what underlies sensory hypersensitivity. Crespi's theory explains repetitive motor behaviors as resulting from a heightened system of pattern generation. A novel part of Crespi's theory, orthogonal to my own proposal, is that upregulating the brain's natural preference for patterns entailed a dialing-down of social information processing, given that social phenomenon are the antithesis of algorithmic patterns. Crespi's ideas about the importance of patterns are consistent with and complementary to my hypothesis that repetitive motor behaviors underwent selection pressure because of the pay-off of inventions.

## Summary

Clare Harrop, a leading researcher of restricted and repetitive behaviors (RRBs), wrote with her colleagues, “.as a field we do not understand what causes RRBs, and this is particularly difficult to ascertain when children are minimally verbal.” (1). The current account is a response to this request for new ideas about the cause of repetitive behaviors. Motor repetitions in autism are an alternative phenotype which has adaptive and functional consequences: fueling trial-and-error tinkering which could lead to inventions, i.e., novel, useful configurations of objects. This hypothesis is consistent with the proposed mechanism of heterochrony (11), and is complementary to other theories which take a strength-based approach to autism (8, 19). This hypothesis helps explain similarities between motor repetitions and circumscribe interests, illuminates parents' observations of their child's motor repetitions [e.g., (30)], and has heuristic value in providing ideas for therapists to introduce flexibility into the motor routines of minimally verbal children.

## Author Contributions

CC-H was responsible for the development of all sections of this manuscript.

## Conflict of Interest

The author declares that the research was conducted in the absence of any commercial or financial relationships that could be construed as a potential conflict of interest.
